# Dataset on performance management systems' design in project-based organizations

**DOI:** 10.1016/j.dib.2019.104185

**Published:** 2019-06-27

**Authors:** Mariska M.G. de Rooij, Remco S. Mannak, Martyna Janowicz-Panjaitan

**Affiliations:** a Vion IM&T BV, Boseind 10, 5328 RM’, Boxtel, the Netherlands; b Department of Organization Studies, Tilburg University, Warandelaan 2, 5038 AB, Tilburg, the Netherlands; c Tilburg School of Economics and Management, Tilburg University, Warandelaan 2, 5038 AB, Tilburg, the Netherlands

**Keywords:** Performance management system, Project-based organization, Fuzzy-set qualitative comparative analysis, Contingency factor

## Abstract

This data article presents the supplementary material for the paper “A configurational explanation for performance management systems' design in project-based organizations” [1]. The article introduces a dataset on 15 project-based organizations (PBOs) in the management consulting industry in the Netherlands. The dataset includes organization-level conditions at PBOs, such as perceived environmental uncertainty, organizational size, innovation strategy, opportunity strategy, and performance management system design. The dataset is prepared for a fuzzy-set Qualitative Comparative Analysis (fsQCA). Combinations of conditions are expected to be related to a mechanistic or an organic performance management system design. This article includes the original dataset with quantitative scores and a qualitative motivation for each score, calibrated data, and fsQCA truth tables.

**Table:** Specifications Table

*Subject area*	Business, management and accounting
*More specific subject area*	Strategy and Management
*Type of data*	Table
*How data was acquired*	Interview, questionnaire, document study
*Data format*	Descriptive, coded, calibrated, processed
*Experimental factors*	The sample includes 15 managers of project-based organizations in the management consulting industry. The authors collected data on perceived environmental uncertainty, organizational size, innovation strategy, opportunity strategy, and the outcome variable performance management system (mechanistic vs. organic).
*Experimental features*	The interviews started with a semi-structured part, to obtain data on the organic and mechanistic controls used in the PBOs performance management system (outcome variable). Thereafter, the interviews continued with a structured part, to determine case scores on each condition by means of validated questionnaires. The final part of the interview was semi-structured (including document study) to validate and motive each case score. Case scores were calibrated and configurations of conditions were presented in fsQCA truth tables.
*Data source location*	The Netherlands
*Data accessibility*	Data is included in this article
*Related research article*	De Rooij, M.M.G., Janowicz-Panjaitan, M, & Mannak, R.S. (2019). A configurational explanation for performance management systems' design in project-based organizations. *International Journal of Project Management,* 37(5), 616-630

**Table:** 

**Value of the data** • The data can be used by managers to support the process of designing a performance management system, based on the combination of organization-level characteristics of their PBO (see Ref. [1] for interpretations). • The data can be used as a benchmark for research on performance management systems of PBOs in other research settings (for a categorization of PBOs, see Ref. [2]) • The data can be used to compare the explanatory power of fsQCA as a method of analysis, relative to other methods, including linear additive approach (for an example, see Ref. [3]) • The data can be used as teaching material for fsQCA (see Refs. [1], [4] for a discussion on the methodology).

## 1 Data

The data was collected in 15 PBOs in the management consulting industry, by means of an interview with a top manager or highly informed middle manager, and a document study (for a case description, see Ref. [1] - Appendix A). Each interview contained a semi-structured part, a structured part, followed by another semi-structured part (for the supplementary interview outline, see Appendix A). The first semi-structured part served to collect data on the mechanistic and organic controls used by the PBO. Performance management system design, the outcome variable, was measured as the PBO's proportion of mechanistic controls relative to their organic controls, as outlined by Ferreira and Otley [5]. The structured part, based on earlier validated questionnaires, served to determine the case scores on each condition. Perceived environmental uncertainty was measured by means of 4 items (7-point Likert scale) developed by Miller [6]. Organizational size reflects the turnover of the PBO. Innovation strategy was measured by means of 3 items adopted from Jansen et al. [7]. Opportunity strategy was measured by means of 3 items derived from Naman and Slevin [8]. The final semi-structured part of the interview served to validate and motivate each case score, as displayed in Table 1. Table 2 presents threshold values for data calibration, while Table 3 features the calibrated data itself. Table 4, Table 5 exhibit the Truth Tables for the mechanistic/organic performance management system design.

**Table 1:** Case scores with motivation.

	PERFORMANCE MANAGEMENT SYSTEM 0-Organic, 100-Mechanistic.		PERCEIVED ENVIRON. UNCERTAINTY 1-Predictable,7-Unpredictable.		SIZE Min € 400K Max € 80M	INNOVATION STRATEGY 1-Exploitative, 7-Explorative.		ENTREPRENEURIAL ORIENTATION 1-Deterministic, 7-Voluntaristic.	
Case 1	40	Organic controls slightly outweigh mechanistic controls, of which the Personnel controls occur most frequently. There is highly reliance on the company's core message and cross-training. Environment is created which facilitates communication and group-driven action. Next to that, financial reports and patriarchal control are used to manage the organization.	4.25	The environment is fairly predictable. Yet, the perception of the environmental uncertainty increased since the business model was changed radically.	€ 1M	6	The organization devotes 30% of its time to R&D, which is very substantial compared to other organizations.	6.5	Firm's core business is helping other organizations change radically. The organization applies the same principles to itself: every few years, it drastically changes its business model, if needed. The firm mainly follows trends from abroad, which they introduce first in the Netherlands. This indicates a voluntaristic orientation.
Case 2	51.43	Organic controls and mechanistic controls pretty much in balance. Most prominent organic controls include sophisticated integrative mechanisms and strategic interactive controls. Administrative use of budget is by far the most important mechanistic control.	3.25	The firm's environment is rather predictable. Its dependence on the environment for input is low and the competitors are known. Predictability is lowered by the firm trying to enter new markets.	€ 6M	4.5	The company is exploring new markets and new customers. It set up a department for market research.	4.5	The organization introduces a new product or radical change in a product almost every year. It is thus quite proactive. At the same time the organization scores rather low on risk-taking.
Case 3	57.89	Has a mechanistic performance management system. Uses narrow controls and targets to control output, results, and behavior. It also uses organic controls like sophisticated integrative mechanism and strategic interactive controls to focus on customer satisfaction and innovative capacity.	5.67	Finds itself in a rather unpredictable market. Product demand changes often and suddenly. Faces “unfair” competition. The only thing that is more predictable is technology.	€ 3M	4.5	Introduces product innovations once or twice a year.	5.33	One of the more progressive organizations in the sector, i.e., voluntaristic orientation. Next to that, organization takes risks and is a market leader in some segments.
Case 4	69.23	Case 4 has clearly a mechanistic performance management system. It relies on weekly statistical rapports on tenders, project resources used and available, invoices, work provision, average work rate, and turnover in total or per person. These statistical controls are complemented with organic controls of which social controls are the most recurring.	2.5	This organization perceives its environment as extremely predictable. The only factor that makes it less predictable is technology as this case operates in an innovative sector.	€ 1.9M	7	This organization systematically provides entirely new services to the customers. They barely repeat projects. Their aim is to learn more from past projects and make more use of proven concepts.	4.67	The organization has introduced a lot of new services and products and is market leader, which results in the fact that other organizations follow case 4. The only thing that lowers the score on voluntarism is that it does not take high risks.
Case 5	43.57	Case 5 has an organic performance management system. It uses mostly social control and personnel control. They do not easily hire and have a long trial period. Working with more than one person on a job is a prerequisite. They complement the organic controls with mechanistic planning.	2	The environment is perceived as very predictable. Everything can be planned and the customers and demands are highly similar.	€ 1M	7	Always concerned with combining different markets in order to make a new product. Claim not to do small improvements, but only radical changes.	2.5	The organization has created its own niche. Thus, it does not actively change its products and services. Risk taking is not necessary. The organization is rather deterministic.
Case 6	36.84	Has a predominantly organic performance management system, with an unconventional form. A broad range of information from the customers and the firm itself is used for decision making.	5	This organization finds itself in a fairly unpredictable environment. The only item that scores a bit higher on predictability is competition.	€ 1M	2	Innovations within this company are mostly exploitative. They stay in the same sector and slowly adapt their services.	3.33	Quite deterministic in orientation. They barely introduce new services and products and do not take risks. The only factor that makes it more voluntaristic is that they stay a bit ahead of the competition.
Case 7	9.5	Rely on organic performance management with focus on the central message, vision and strategy at its core. They believe in the competences of the professionals; targets for the professionals whereby earnings largely accrue to the person who generated them it in the first place.	4.5	This organization struggles to find human resources and perceives this aspect of their environment as very unpredictable. For the rest environment is quite predictable as business is very specialized and the sector is quite small.	€ 1M	1	This organization follows the product changes of their supplier and only incrementally adapts the product to the customer's needs.	3.33	This organization is rather deterministic. Except for the fact that competition watches them as the new comer in the field, they do not actively create any new opportunities.
Case 8	29.63	This organic performance management system is largely focused on getting the right people on board, communicating the core value and creating a group of employees that supports each other. Within those boundaries employees get the freedom and are controlled only on output and results.	3.5	The environment is perceived as pretty predictable. The only factor that makes it less predictable are the human resources. The case experiences it as rather hard to find the right employees.	€ 1.4M	5	This organization aims to develop innovative solutions for its customers. Despite that focus, their strategy also has exploitative elements, but they try to be explorative when customers ask for it.	3.5	Besides parts of projects being reused, in the last 5 years the overall range of services changed often. This organization also takes some risk. The one thing that makes this organization less voluntaristic is that it tries to avoid competition.
Case 9	66.67	This predominantly mechanistic performance management system departs from communicating the vision and mission, knowing the key success factors and then translating those into measurable features. Those measures are designed together with the employees and employees' rewards are based upon the evaluation on those measures.	5	The environment is perceived as uncertain. The project resources, customer demands, and technology are rather uncertain. The only thing that is certain is the competition: this firm has a clear view of the competitors and what they do.	€ 80M	3	This organization adapts its products to customer demands. The products are provided by a supplier and the organization customizes those to the customer needs.	5.83	This organization has a rather voluntaristic strategy. It takes high risks and is one of the organizations that is a frontrunner in the sector. Next to that, they had many product changes, although driven by their supplier.
Case 10	54.16	Mechanistic performance management system that incorporates several organic controls. The firm combines severe assessment during the application procedure with continuous training after employees are hired. They create a year plan for the company in which the individual departments independently create their policy. Their results are then monitored using turnover, acquisition and billable hours.	2.12	The environment is perceived as pretty certain. The only factor that makes it less certain is the new upcoming trend of self-employed professionals.	€ 50M	3.5	This organization collaborates often with partners to help clients from other sectors and clients with new issues. The projects build on standard products that are adapted to the client needs.	5.5	This organization introduces large changes in the product and service range. These changes are client-driven. The organization executes projects both with high and low risks.
Case 11	36.11	Continuous training and intensive coaching for the professionals is key in this organic performance management system. Employees are included in developing the company which creates involvement. At the same time, projects are managed and controlled with financial and accounting controls.	2.33	The environment is perceived as pretty certain. The only factor that makes it less certain is the new upcoming trend of self-employed professionals.	€ 48M	2	This organization is always looking for ways to improve its service. While they aim to recombine old services into new ones, most changes involved involve small modifications to existing services.	6	This organization radically changed its business model over the past years. Hence, they acted highly voluntaristicly. They also take relatively large risks. The organization does not take into account whether it follows or leads the competitors.
Case 12	29.63	The service level is the most important factor in this organic performance management system. Everything that contributes to that goal is encouraged. The employees bear the responsibility for achieving the goal and are supported through training, coaching and knowledge clusters.	2.38	This organization finds itself in a certain environment. The only aspect that makes it less predictable is the technology in the long run.	€ 27M	2	The innovations are mainly focused on adapting the service to the customer. The organization does not engage in radical changes itself. The supplier is the one who delivers the radical changes.	3.66	This organization is more on the deterministic side of the scale. The firm has not changed their service range dramatically over the last period. Although they are the ones followed by their competitors, they have products with both high risks and low risks.
Case 13	57.69	This mechanistic performance management system is driven by output and result control. The firm monitors employees in terms of generated turnover, achieved impact and customer portfolio. Next to that, they manage by communicating the company's vision and organizing monthly meetings to encourage knowledge sharing.	4.33	There are both predictable and unpredictable factors in this organization's environment. The demand and technology are uncertain, but resources and competition are more on the predictable side.	€ 6M	5	This organization tries to come up with many new services. Although they do not perceive them as radical (anymore), the examples indicate explorative innovation.	6	In the past 6 years, this organization changed its business model and became a front runner in the sector, which makes it voluntaristic. Furthermore, they take quite high risks, although keeping them within the possibilities of the organization.
Case 14	43.75	This organic performance management system is relatively small. The firm encourages intense contacts among the employees and knowledge exchange. Additionally, it sets turnover targets, the progress on which is monitored every 3 months.	3	The environment is quite certain, but the economic situation makes it somewhat more uncertain.	420K	1.5	Firm's services change incrementally. In the future it aims to become a bit more radical, but for now the change in services is slow.	3	The accumulated slow changes in the services resulted in a substantial change in services over the past 5 years. However, all projects have low risks.
Case 15	80	This mechanistic performance management system is primarily build around accounting controls such as financial reports. The firm expects its employees to report in a strictly predefined format and check those reports.	6	The resources availability and the demand are highly unpredictable. The factor that makes the environment somewhat more predictable are the type of questions that customers ask.	420K	1	This organization is specialized in one service and customizes this service to various clients, without radically changing the service.	2	The strategy is mainly deterministic, even though the type of clients changed somewhat over the last years. Furthermore the risks are low and competition is not very relevant.

**Table 2:** Threshold values.

	Full non-membership score (0)	Case-crossover point (0.5)	Full membership score (1)
Performance Management System	20%	50%	80%
Perceived Environmental Uncertainty	2	4	6
Size	2,000,000	10,000,000	50,000,000
Innovation Strategy	2	4	6
Entrepreneurial Orientation	2	4	6

**Table 3:** Calibrated data.

	Performance Management System	Perceived Environ. Uncertainty	Size	Innovation Strategy	Entrepreneurial Orientation
Case 1	0.27	0.59	0.03	0.95	0.98
Case 2	0.54	0.25	0.18	0.68	0.68
Case 3	0.69	0.92	0.07	0.68	0.88
Case 4	0.87	0.10	0.05	0.99	0.73
Case 5	0.34	0.05	0.03	0.99	0.10
Case 6	0.21	0.82	0.03	0.05	0.27
Case 7	0.02	0.68	0.03	0.01	0.27
Case 8	0.12	0.32	0.04	0.82	0.32
Case 9	0.84	0.82	0.99	0.18	0.94
Case 10	0.60	0.06	0.95	0.32	0.90
Case 11	0.20	0.08	0.95	0.05	0.95
Case 12	0.12	0.08	0.78	0.05	0.38
Case 13	0.68	0.62	0.18	0.82	0.95
Case 14	0.35	0.18	0.03	0.02	0.18
Case 15	0.95	0.95	0.03	0.01	0.05

**Table 4:** Truth table *Mechanistic* performance management system.

Perceived Environ. Uncertainty	Size	Innovation Strategy	Entrepreneurial Orientation	Number of Cases	Performance Management System	Raw Consistency
1	1	0	1	1	1	0.994
0	0	1	1	2	1	0.837
1	0	1	1	3	1	0.818
1	0	0	0	3	0	0.632
0	1	0	1	2	0	0.618
0	1	0	0	1	0	0.617
0	0	0	0	1	0	0.608
0	0	1	0	2	0	0.564

**Table 5:** Truth table *Organic* performance management system.

Perceived Environ. Uncertainty	Size	Innovation Strategy	Entrepreneurial Orientation	Number of Cases	Performance Management System	Raw Consistency
0	1	0	0	1	1	1.000
0	0	0	0	1	1	0.928
0	0	1	0	2	1	0.852
0	1	0	1	2	1	0.850
1	0	1	1	3	0	0.766
1	0	0	0	3	0	0.733
0	0	1	1	2	0	0.702
1	1	0	1	1	0	0.607

## 2 Experimental design, materials and methods

To facilitate the educational use of the data and potential replication studies, the data has been calibrated [9] into fuzzy scores in the interval between 0 and 1. Defining threshold values is key for determining the degree to which a case belongs to a condition, fully in (1), fully out (0) or maximal ambiguous (0.5 – case-crossover point). Based on the case score motivations and the scales used to measure each variable, we determined initial threshold values. We verified the threshold values by means of a cluster analysis (for further details, see Ref. [1]). The threshold values are presented in Table 2. The data reveals two clusters of PBOs, one cluster of 8 PBOs using predominantly organic controls (on average 66.4% organic controls; min. 56.3% max. 90.5%), and the other cluster of 7 PBOs using predominantly mechanistic controls (on average 62.4% mechanistic controls, min. 51.4% max. 80%). The calibrated data is displayed in Table 3.

The calibrated scores allow for conducting fsQCA on the combinations of conditions (pathways) that in conjunction either relate to organic or mechanistic performance management system design. In short, fsQCA examines *combinations* of conditions leading to a specific outcome, instead of examining conditions in isolation. It allows for different pathways to the same outcome (equifinality), as well as distinct pathways to opposite outcomes (asymmetry). For an elaborated discussion on fsQCA as methodology, see Refs. [1], [4]. For identifying configurations of conditions, a Fuzzy Truth Table Algorithm was used. The truth tables (Table 4, Table 5) were derived with the fs/QCA software, by using a consistency cutoff value of 0.8 and a minimum of 1 case per solution term. The actual solution terms are presented and discussed in De Rooij et al. [1]. They can be replicated by means of the ‘standard analysis’ option of the fs/QCA software, having all prime implicants marked. The fsQCA reveals a transparent two-path solution per outcome (mechanistic/organic performance management system design), which makes the data particularly useful for educational purposes.
