# Supplementary material for: Dataset on performance management systems' design in project-based organizations
Source: Data Brief. 2019 Jun 27;25:104185. doi: 10.1016/j.dib.2019.104185 (PMC6661388; doi:10.1016/j.dib.2019.104185)
Supplement: Multimedia component 1 [file mmc1.docx]

**Interview outline “A configurational explanation for performance management systems' design in project-based organizations”**

## **I. General respondent information**

1. What is your function within the organization?
2. What is your educational and professional background?
3. How long do you work at this organization?

## **II. General organization information**

1. What is the number of employees at your organization?
2. What year was the organization founded?
3. How many projects (approximately) does the organization run at the moment?
4. How big are the projects on average? (in terms of scale)

## **III. Management control system**

(Ferreira and Otley, 2009)

1. What is the vision and mission of the organization and how is this brought to the attention of managers and employees? What mechanisms, processes, and networks are used to convey the organization’s overarching purposes and objectives to its members?
2. What are the key factors that are believed to be central to the organization’s overall future success and how are they brought to the attention of managers and employees?
3. What is the organization structure and what impact does it have on the design and use of management systems? How does it influence and how is it influenced by the strategic management process?
4. What strategies and plans has the organization adopted and what are the processes and activities that it has decided will be required for it to ensure its success. How are strategies and plans adapted, generated and communicated to managers and employees?
5. What are the organization’s key performance measures deriving from its objectives, key success factors, and strategies and plans? How are these specified and communicated and what role do they play in performance evaluation?
6. What level of performance does the organization need to achieve for each of its key performance measures (identified in the above question), how does it go about setting appropriate performance targets for them, and how challenging are those performance targets?
7. What processes, if any, does the organization follow for evaluating individual, group, and organizational performance? Are performance evaluations primarily objective, subjective or mixed and how important are formal and informal information and controls in these processes?
8. What rewards — financial and/or non-financial — will managers and other employees gain by achieving performance targets or other assessed aspects of performance(or, conversely, what penalties will they suffer by failing to achieve them)?

## **IV. Perceived environmental uncertainty**

(Adapted from Miller, 1993)

In this section, we would like you to describe the environment in which your company operates. In the primary industry and country where you work, evaluate the aspects of your environment. Indicate if the factors are easy or difficult to predict. 1 -Easy to predict, 7-Unpredictable

| 1. How predictable are the resources and services used by your company? So the availability of trained labor, problems with labor and union problems, the quality of inputs, raw material and components, the prices of inputs, and raw materials and components. |
| --- |
| 1. How predictable are the product market and demand? Keep in mind the predictability of client preferences, product demand, availability of substitute products and the availability of complementary products. |
| 1. How predictable is the competition? Take in to consideration the predictability of changes in competitors’ prices, changes in the markets served by competitors, changes in competitors’ strategies, entry of new firms into the market and domestic and foreign competitors. |
| 1. How predictable is the technology in your industry? Think about the predictability of product changes, changes in product quality, new product introductions and changes in the production process? |

## **V. Strategy: exploratory versus exploitative**

(Adapted from Jansen et al., 2006)

In this section, we would like you to describe the strategy your company pursues. Indicate if the statements are applicable to your organization.

| 1. How much does your organization focus on exploratory innovation? So does your organization accept demands that go beyond existing products and services, invent new products and services, experiment and commercialize completely new products or services, and frequently use new opportunities in new markets or new distribution channels? |
| --- |
| 1 –Never , 7- All innovation |
| 1. How much does your organization focus on exploitative innovation? Think about if your organization frequently refines the provision of existing products and services, implements small adaptations, introduces improved but existing product and services, improves the provision’s efficiency of products and services, increases economies of scale in existing markets and expands services for existing clients. |
| 1 –Never , 7- All innovation |
| 1. Is your organization more focused on exploitative or explorative innovation? |
| 1 – Exploitative, 7- Explorative |

## **VI. Strategy: Deterministic versus voluntaristic**

(Naman and Slevin, 1993)

In this section we would like to ask you describe the strategy of your company. Please indicate to which statement you agree more. 1 – first statement, 7 second statement

| 1. In the past 5 years… | |
| --- | --- |
| Did your organization not market new products or services, change only the products and services incrementally and have a strong emphasis on marketing on tried and true products or services. | Did your organization market many new lines of products or services, change product or service lines dramatically or have a strong emphasis on R&D, technological leadership and innovations? |
| 1. How does your organization deal with competition? | |
| Do you generally respond to competitors, almost never introduce innovations and avoid competitive clashes? | Or do you typically initiate actions to which competitors respond, often introduce innovations first and prefer to enter the competition? |
| 1. In general… | |
| This organization has strong proclivity for low risk projects, believes it is best to explore projects gradually via cautious, incremental behavior and typically adopts a cautious, "wait and see" posture in order to minimize the probability of making costly decisions. | A strong proclivity for high risk projects, believes bold and wide-ranging acts are necessary to achieve the firm's objectives and typically adopts a bold, aggressive posture in order to maximize the probability of exploiting potential opportunities. |
|  | |
